# Metabolic Therapy for Temporal Lobe Epilepsy in a Dish: Investigating Mechanisms of Ketogenic Diet using Electrophysiological Recordings in Hippocampal Slices

**DOI:** 10.3389/fnmol.2016.00112

**Published:** 2016-11-01

**Authors:** Masahito Jr. Kawamura, David N. Ruskin, Susan A. Masino

**Affiliations:** ^1^Department of Pharmacology, Jikei University School of MedicineTokyo, Japan; ^2^Department of Psychology and Neuroscience Program, Trinity CollegeHartford, CT, USA

**Keywords:** ketone bodies, adenosine receptors, pannexin channels, ATP-sensitive potassium channels, vesicular glutamate transporter, temporal lobe epilepsy

## Abstract

The hippocampus is prone to epileptic seizures and is a key brain region and experimental platform for investigating mechanisms associated with the abnormal neuronal excitability that characterizes a seizure. Accordingly, the hippocampal slice is a common *in vitro* model to study treatments that may prevent or reduce seizure activity. The ketogenic diet is a metabolic therapy used to treat epilepsy in adults and children for nearly 100 years; it can reduce or eliminate even severe or refractory seizures. New insights into its underlying mechanisms have been revealed by diverse types of electrophysiological recordings in hippocampal slices. Here we review these reports and their relevant mechanistic findings. We acknowledge that a major difficulty in using hippocampal slices is the inability to reproduce precisely the *in vivo* condition of ketogenic diet feeding in any *in vitro* preparation, and progress has been made in this *in vivo/in vitro* transition. Thus far at least three different approaches are reported to reproduce relevant diet effects in the hippocampal slices: (1) direct application of ketone bodies; (2) mimicking the ketogenic diet condition during a whole-cell patch-clamp technique; and (3) reduced glucose incubation of hippocampal slices from ketogenic diet–fed animals. Significant results have been found with each of these methods and provide options for further study into short- and long-term mechanisms including Adenosine triphosphate (ATP)-sensitive potassium (K_ATP_) channels, vesicular glutamate transporter (VGLUT), pannexin channels and adenosine receptors underlying ketogenic diet and other forms of metabolic therapy.

## Hippocampus: A Key Brain Region to Investigate Ketogenic Diet Mechanisms

The hippocampus is well-known as a brain area involved in learning and memory and also as the key region underlying the form of epilepsy known as mesial temporal lobe epilepsy. The temporal lobe refers to the ventrolateral middle part of cerebral cortex and abnormal neuronal discharge or a lesion affecting this lobe causes seizures (Gastaut, 1973). There are two main types of temporal lobe epilepsy classified by the epileptic focus: mesial and lateral. The epileptic focus of mesial temporal lobe epilepsy is the hippocampus, amygdala or parahippocampal gyrus and the focus of lateral temporal lobe epilepsy is in neocortex. Over 80% of patients with temporal lobe epilepsy have the mesial form (Schramm et al., 2001; Quarato et al., 2005) and it is often resistant to pharmacological treatment. The typical symptom of mesial temporal lobe epilepsy is complex partial seizures, which have a high probability of an accompanying characteristic aura. For an individual patient the aura may present as epigastric discomfort sometimes described as nausea, or psychiatric symptoms including fear. Complex partial seizures often begin with arrest of motor activities or staring. Autonomic motor behaviors are usually oroalimentary automatisms or complex automatisms. Dystonic posturing lasting for 1–2 min often occurs involving the arm contralateral to the ictal discharge (Engel, 2001). In a majority of patients, mesial temporal lobe epilepsies are associated with hippocampal sclerosis (Watson, 2003), which is atrophy with global gliosis and loss of CA1 and/or CA3 pyramidal neurons in the hippocampus (Thom, 2009).

The basic structure of hippocampus is simple. It includes principal cells (granular cells of dentate gyrus and CA1-4 pyramidal neurons) and surrounding interneurons. The principal cells form an excitatory circuit which is modulated by inhibitory interneurons. Notably, CA3 pyramidal neurons are connected to each other by excitatory recurrent collaterals. Thus, the hippocampal circuit is regulated by a balance between excitation from recurrent collaterals and inhibition from interneurons. When the balance collapses, the hippocampal circuit becomes hyper excitable and susceptible to seizures. Therefore, atrophy in the hippocampus is thought to be one of the main focuses of mesial temporal lobe epilepsy and excision of hippocampal sclerosis with selective amygdalohippocampectomy successfully improves ~70% of surgical patients (Wiebe et al., 2001; Paglioli et al., 2006). Unfortunately, even though a majority experience significant improvement after surgery, at least half of patients relapse and do not experience permanent and complete seizure control (McIntosh et al., 2004; de Tisi et al., 2011; Najm et al., 2013). Based on clinical and experimental evidence, the hippocampus is a good experimental target for investigating epileptogenesis and therapeutic interventions for temporal lobe epilepsy.

The ketogenic diet was designed in the 1920s to treat epilepsy by mimicking the metabolic changes induced by fasting (Wilder, 1921). Ketogenic diet is effective against many types of seizures. It is used more frequently against generalized seizures such as myoclonic, atonic and absence seizures. However, it is also reported that ketogenic diet has been used successfully to treat focal seizures such as simple and complex partial seizures as effectively as generalized seizures [Freeman et al., 1998; Maydell et al., 2001; but one study reported that the ketogenic diet was less effective in patients with epileptiform discharges in the temporal region (Beniczky et al., 2010)]. Despite almost 100 years of clinical use, however, the mechanisms underlying the success of ketogenic diet therapy are not well understood. In recent decades, ketogenic diet has increasingly been noted as a useful therapy for medically refractory epilepsy in adults (Sirven et al., 1999; Mosek et al., 2009) and children (Hallböök et al., 2007). Patients with temporal lobe epilepsy are well-known to be frequently resistant to antiepileptic drugs (Wiebe and Jette, 2012). As mentioned above, the first choice of treatment for medically refractory mesial temporal lobe epilepsy associated with hippocampal sclerosis is surgery (anterior temporal lobectomy or selective amygdalohippocampectomy) because of good therapeutic outcomes (Tanriverdi et al., 2008). For temporal lobe epilepsy patients who are not good candidates for surgery, however, ketogenic diet is one of the therapeutic options (Ray and Wyllie, 2005; Klein et al., 2010). Thus, a natural question is how the ketogenic diet produces its beneficial effects in temporal lobe epilepsy. Broad reviews on the diet’s mechanisms are available (Lutas and Yellen, 2013; Rogawski et al., 2016); here, we focus on work from multiple laboratories studying ketogenic diet’s antiseizure mechanisms using acute hippocampal slice preparations.

## Advantages of the Hippocampal Slice Preparation in Studying Antiepileptic Mechanisms of Ketogenic Diet

Because epilepsy is caused by abnormal neuronal discharges in the brain, electrophysiological measurements are the most direct and useful approach for researching epilepsy and its treatments. There are two approaches for electrophysiological recordings of any brain region: *in vivo* and *in vitro*. *In vivo* electrophysiological recording of hippocampus is usually done by extracellular recording of electrically-evoked activity (Stewart and Reid, 1993), or continuous recording of spontaneous field activity (Li et al., 2008), single-cell intracellular sharp electrodes (Henze and Buzsáki, 2001) or multiple unit activity (Lin et al., 2006); these preparations can be acute or chronic. The technique for *in vivo* patch-clamp recording was also developed recently (Pernia-Andrade and Jonas, 2014). *In vitro* electrophysiological recording is done using single-cell intracellular sharp electrodes (Abe and Ogata, 1981) or patch-clamp electrodes (Kawamura et al., 2004), or extracellular field recording with single electrodes (Masino and Dunwiddie, 1999) or electrode arrays (Knowles et al., 1987) using acute slices of hippocampus. Compared with *in vivo* hippocampal recordings, the advantages of hippocampal slices are several-fold: (1) Ease of use and tissue access: acute brain slices must be maintained by perfusion with oxygenated artificial cerebrospinal fluid (Sakmann et al., 1989). Continuous perfusion allows for changing the extracellular fluid, making it easy to apply and wash out agonists and/or antagonists of various proteins such as ion channels, receptors and transporters. Furthermore, it is easy to examine in detail the functional mechanisms and dynamics of neuronal activity; (2) Efficient use of resources: we usually make 3–6 brain slices from one rodent, potentially obtaining 3–6 recordings, and thus allowing us to reduce the number of animals used; (3) History: a huge number of electrophysiological experiments have been done using hippocampal slice preparations in the last half-century. Several methods for causing seizure-like bursting *in vitro* have been used in the hippocampal slice preparation including electrical kindling (Sayin et al., 1999), kainic acid treatment (Congar et al., 2000; Smith and Dudek, 2001), inhibition of GABA receptors (Köhling et al., 2000; Stafstrom et al., 2009), inhibition of potassium ion channels (Stafstrom et al., 2009) and neuronal hyperexcitability by high extracellular potassium concentrations (Congar et al., 2000; Stafstrom et al., 2009) or low extracellular magnesium concentrations (Dulla et al., 2005; Kovács et al., 2005); and (4) Potential use of human tissue: experimental techniques from rodent hippocampal slice preparations are also applicable to acutely resected hippocampal tissue obtained from patients with surgically approachable epilepsy (Schroder et al., 2000). All these approaches support the use of hippocampal slice preparations to elucidate epileptic mechanisms.

Among these advantages lurk some disadvantages. One unavoidable pitfall of *in vitro* recording is that the environment of acute brain slice preparations is inherently different from *in vivo* condition. Cutting brain tissue causes acute traumatic injury such as excitatory GABA signaling caused by increased intracellular chloride concentration in acute hippocampal preparations (Dzhala et al., 2012). It is known that gliosis occurs in the hippocampal slice cultures (Lossi et al., 2009). Recently, it has been reported that the early stage of reactive gliosis already occurs in acute hippocampal slice preparations (Takano et al., 2014). We usually cut slices at 300–500 μm, necessarily limiting neuronal networks and three-dimensional morphologies to this thickness. Also, artificial cerebrospinal fluid does not and cannot reproduce actual cerebrospinal fluid exactly. Thus, results from *in vitro* hippocampal slice preparations should be confirmed by *in vivo* electrophysiological recordings or behavioral tests as much as possible. For that reason both *in vivo* and *in vitro* electrophysiological recordings are useful and both are essential for epilepsy research.

**Figure 1:**
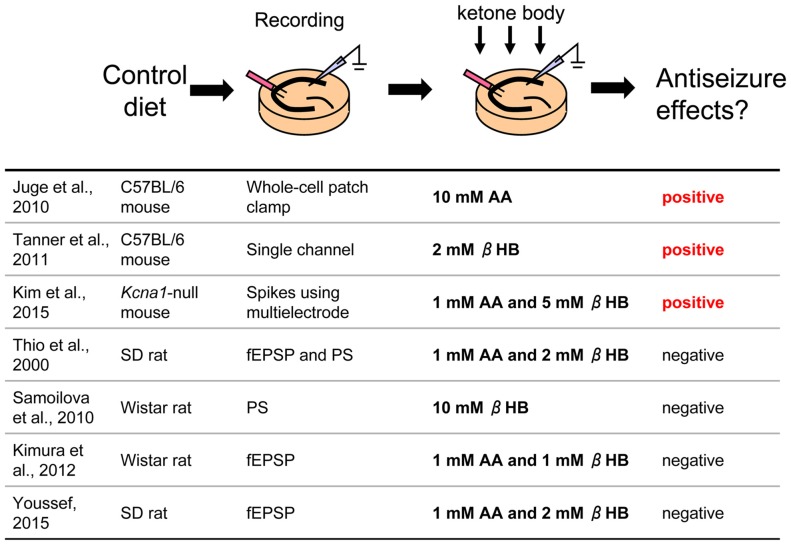
**Direct application of ketone bodies to hippocampal slices From control diet-fed animals.** Reference list is shown at bottom. Three of seven references report positive effects for antiseizure with direct application of ketone bodies. AA, acetoacetate; βHB, β-hydroxybutyrate; fEPSP, field postsynaptic potential; PS, population spike.

**Figure 2:**
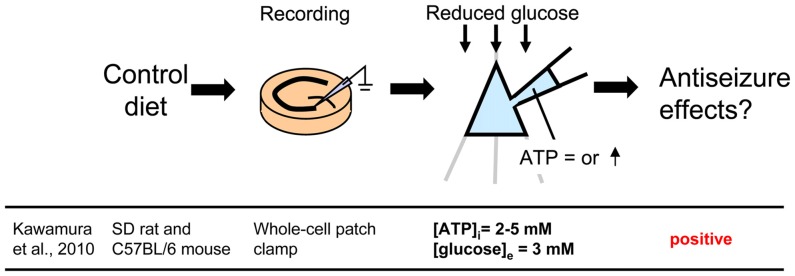
**Controlling glucose and adenosine triphosphate (ATP) levels by using whole-cell patch clamp recording of a single cell in a hippocampal slice from control diet-fed animals.** To mimic the ketogenic diet milieu, extracellular glucose is reduced and intracellular ATP is increased. Reference is shown at bottom. [ATP]_i_, intracellular ATP concentration; [glucose]_e_, extracellular glucose concentration.

**Figure 3:**
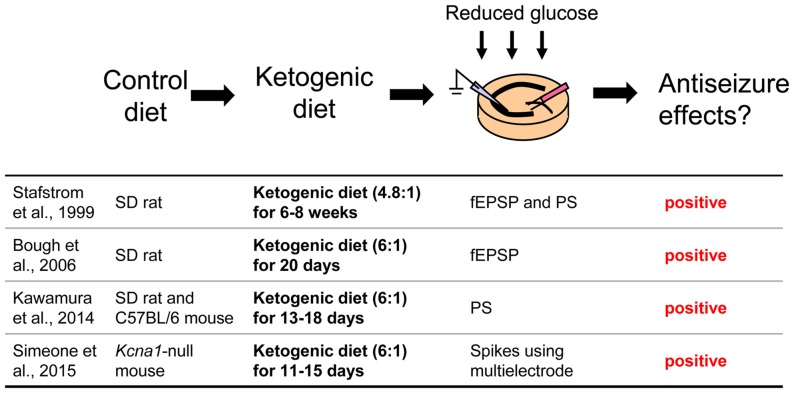
**Acute hippocampal slices from ketogenic diet-fed animals.** Extracellular glucose is reduced during incubation and recording to maintain the *in vivo* effect of the ketogenic diet. Reference list is shown at bottom. fEPSP, field postsynaptic potential; PS, population spike. Ketogenic diet (4.8:1) or (6:1) means fat: [protein + carbohydrate] ratio.

Ketogenic diet therapy presumably alters aspects of blood and cerebrospinal fluid to produce an anticonvulsant effect; in making and supporting brain slices, it is necessary to replace blood and cerebrospinal fluid with a bathing solution. Thus, the major difficulty in using acute hippocampal slices for ketogenic diet research is the inability to precisely reproduce or maintain the metabolic condition induced by diet therapy in this *in vitro* preparation. The lack of a standard protocol for a “diet in a dish” is evident in the diversity of experimental procedures, the prevalence of mixed results and the relative dearth of studies given the importance of delineating the key mechanisms underlying an enduring and successful treatment for a common and challenging neurological condition. *In vivo* recording clearly does not have this problem because it uses the whole body of experimental animals and diet-altered metabolism is maintained (Koranda et al., 2011; Masino et al., 2011). Several special strategies have been implemented for examining mechanisms of the ketogenic diet using hippocampal slice preparations; we focus on reviewing three of these approaches (Figures 1–3).

## Three Strategies for Investigating Ketogenic Diet Mechanisms in Hippocampal Slices

### Direct Application of Ketone Bodies to Hippocampal Slices From Control Diet-Fed Rodents

The ketogenic diet was developed to mimic fasting, which alleviates epileptic seizures (Wilder, 1921). Ketogenic diet feeding increases ketone bodies (β-hydroxybutyrate (βHB), acetoacetate (AA), acetone) to usually over 1 mM in blood in humans (Bergqvist et al., 2005; Than et al., 2005) and rodents (Hartman et al., 2008; Linard et al., 2010) synthesized from free fatty acids in the liver (Masino and Rho, 2012) and then used for energy in the brain instead of glucose (Masino et al., 2009). Chronic ketosis (increased levels of ketone bodies) is the eponymous metabolic hallmark of the ketogenic diet. Therefore, one approach to reproducing a ketogenic diet in a hippocampal slice is a direct application of ketone bodies to determine if and how ketone bodies modulate neuronal activity directly.

In this paradigm, hippocampal slices are taken from control diet-fed animals and dissolved ketone bodies are applied in an extracellular solution such as artificial cerebrospinal fluid (Figure 1). Slice preparation protocols, electrophysiological recording methods, rodent model (different strains of rats or mice), and mixtures of ketone bodies can be found among the laboratories. Not surprisingly, results among the studies were mixed.

A number of studies found negative results. Thio et al. (2000) reported that direct application of ketone bodies had no effect on synaptic activity in acute hippocampal slices from Sprague-Dawley (SD) rats. They recorded evoked field excitatory postsynaptic potentials (fEPSP) and population spikes (PS) in the CA1 region stimulated by Schaffer collateral fibers and applied mixed ketone bodies (1 mM AA and 2 mM βHB) to the slices. A 20 min application of ketone bodies did not change either fEPSP slope or PS amplitude. They also recorded potassium channel blocker 4-aminopyridine-induced epileptiform discharges from the dentate granule cell layer and CA3 region and reported that application for 105 min did not change the frequency or duration of these ictal events. Kimura et al. (2012) also reported that application of mixed ketone bodies (1 mM each AA and βHB) for 20 min did not change fEPSP slope and an 80 min application did not change the high-frequency tetanic stimulation-induced long-term potentiation (LTP) recorded from CA1 region in acute hippocampal slices from Wistar rats. Similar results were reported that mixed ketone bodies (1 mM AA and 2 mM βHB) had no effect on CA1 region synaptic transmission or theta burst-induced LTP in SD rat acute hippocampal slices (Youssef, 2015). A unique approach was used by Samoilova et al. (2010). They made organotypic hippocampal slices which were cultured with low glucose and 10 mM βHB medium for at least 3 days. This chronic *in vitro* ketosis, however, did not alleviate intrinsic or induced epileptiform discharges (but was neuroprotective). All of these studies concluded that ketone bodies do not directly affect synaptic transmission, seizure-like activity or LTP in the rat hippocampal slice.

Other studies, however, have found positive results and even revealed new mechanisms. Juge et al. (2010) made acute hippocampal slices from C57BL/6 mice and incubated the slices with 10 mM AA for over 2 h, after which they recorded EPSPs from CA1 pyramidal neurons using whole-cell patch clamp. Frequency and amplitude of miniature EPSPs (mEPSP) from AA-incubated slices were significantly reduced compared with control slices. Ketone bodies inhibited valinomycin-evoked glutamate uptake by the purified vesicular glutamate transporter (VGLUT), suggesting that ketone bodies inhibit synaptic transmission with reduction of glutamate release via direct ketone body-induced suppression of glutamate uptake into vesicles. Importantly, they also investigated the behavioral effects of ketone bodies. Seizures in Wistar rats induced by intrahippocampal 4-aminopyridine were moderated by intrahippocampal 10 mM AA, both infused by microdialysis. These results clearly show that direct application of ketone bodies modulates synaptic transmission in hippocampal slices and reduces seizure activity *in vivo* (Juge et al., 2010). In addition, Tanner et al. (2011) recorded single channel activity from dentate granule neurons after incubating acute hippocampal slices from C57BL/6 mice with 2 mM βHB for over 40 min. Preincubation with this ketone body increased steady-state and stimulus-elevated open probability of Adenosine triphosphate (ATP)-sensitive potassium (K_ATP_) channels, which contribute to the slow afterhyperpolarization after action potential bursts to modulate spontaneous firing, suggesting that direct ketone body-mediated opening of K_ATP_ channels in dentate granule neurons may act as a seizure gate in the hippocampus. Similar results were reported from neurons of the substantia nigra in coronal midbrain slices of rats and mice from same laboratory (Ma et al., 2007). Kim et al. (2015) recorded from organotypic hippocampal slices which were cultured with 5 mM βHB and 1 mM AA medium for 2 weeks. They used *Kcna1*-null mice (C3HeB/FeJ background) lacking voltage-gated potassium (K_v_1.1) channels, which is thought be a model for several types of epilepsy including human temporal lobe epilepsy. Extracellular multielectrode array recordings showed spontaneous seizure-like events in organotypic hippocampal slice cultures from *Kcna1*-null mice. The application of ketone bodies for 2 weeks attenuated the seizure-like events in the mutant tissue. They also applied 5 mM βHB *in vivo* using subcutaneously implanted osmotic minipumps to *Kcna1*-null mice and reported that administration of this ketone body reduced the number of seizures (Kim et al., 2015). In typical studies, slice preparations are maintained by higher extracellular glucose concentration than is physiological; up to 25–30 mM extracellular glucose concentration for acute hippocampal slices (Bischofberger et al., 2006) and 10–12 mM extracellular glucose for hippocampal slice cultures [Galow et al., 2014; but electrophysiological recording can be done in 5–10 mM glucose (Schneider et al., 2015)]. Since it is reported that complete replacement from glucose to ketone bodies decreases neuronal activity (Arakawa et al., 1991; Wada et al., 1997), all experiments for direct application of ketone bodies were done by adding ketone bodies to the usual *in vitro* glucose concentration for slice preparations. Experiments using purified VGLUT by Juge et al. (2010) were done with glucose-free conditions and the results showed the direct effect of ketone bodies clearly. Ma et al. (2007) compared the effect of ketone body application ranging between 12 mM and 5 mM extracellular glucose concentration in coronal midbrain slices and found the effect was not changed by reduced glucose. These reports suggest that the effects of ketone bodies might not be correlated with glucose concentration.

In sum, several studies have used direct application of ketone bodies in hippocampal acute slices or organotypic cultures, and both positive and negative results have been found. Negative and positive studies used rats and mice, respectively, so a simple explanation is that the discrepancy arises from species differences. This seems unlikely because ketogenic diet is known to reduce behavioral seizures in both rats (Appleton and DeVivo, 1974; Hori et al., 1997; Bough et al., 2002, 2006; Zhao et al., 2004), and mice (Uhlemann and Neims, 1972; Rho et al., 1999; Noh et al., 2003; Hartman et al., 2008; Kwon et al., 2008; but see Linard et al., 2010). The methods for applying ketone bodies varied in these reports including concentration of ketone bodies, time for application and application pathway (perfusion or preincubation) and these might contribute to inter-study variation. Aligning technical details for direct application of ketone bodies may be useful for finding common mechanisms.

### Changing Intracellular ATP and Extracellular Glucose With Whole-Cell Patch Clamp to Mimic Ketogenic Diet

**Figure 4:**
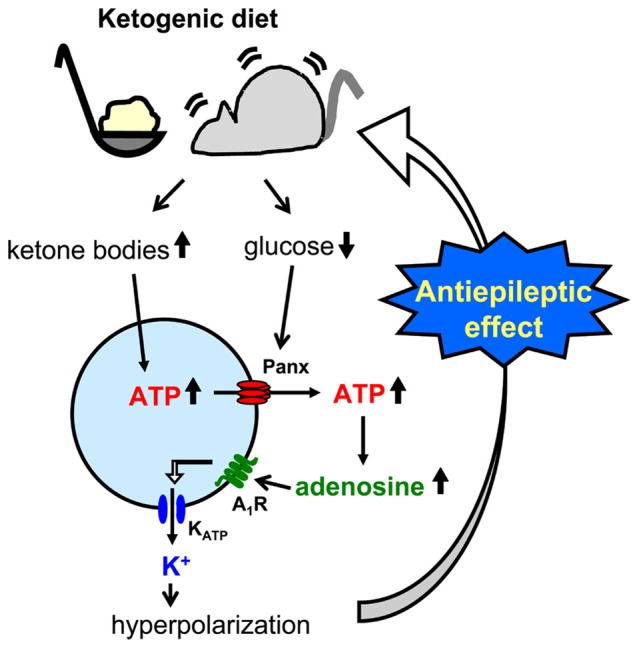
**Schematic of pannexin-1 channel-adenosine A_1_ receptor-K_ATP_ channel autocrine regulation in CA3 pyramidal neurons and relationship to ketogenic diet.** Ketogenic diet-fed animal increases ketone body levels and reduces glucose levels. Ketone bodies might increase intracellular ATP concentration, and increased ATP is released to the extracellular space through reduced glucose-induced opening of pannexin-1 channels (panx). After breakdown to adenosine by nucleotidases, activated adenosine A_1_ receptors (A_1_R) open ATP-sensitive potassium (K_ATP_) channels which hyperpolarizes CA3 pyramidal neurons. This hyperpolarization reduces neuronal hyperexcitability and causes the ketogenic diet’s antiseizure effects.

A less common approach mimics the altered metabolism found during ketogenic diet treatment using single-cell patch clamp recording. Fasting and ketogenic diet are thought to cause anticonvulsant effect by changing brain metabolism, and this approach attempts to mimic a key metabolic “end point.” The other metabolic hallmark (besides ketosis) of the ketogenic diet is a stable, mild hypoglycemia in humans (Huttenlocher, 1976; Noakes et al., 2006; Nuttall et al., 2015) and rodents (Bough et al., 2006). It is also reported that ketogenic diet decreases glucose concentration in the hippocampal extracellular fluid by 30% compared to control diet measured by *in vivo* microdialysis in mice (although lactate did not change; Samala et al., 2011). Interestingly, plasma glucose level correlates with the antiepileptic effect of the ketogenic diet (Mantis et al., 2004), indicating that extracellular glucose is mechanistically relevant. Intracellular conditions are also thought to be changed by ketogenic diet-altered metabolism and product major goal of brain energy metabolism is the generation of sufficient ATP. Several studies have reported that ketogenic diet increases brain ATP levels in humans (Pan et al., 1995) and rodents (DeVivo et al., 1978; Nakazawa et al., 1983; Nylen et al., 2009), thought to be caused by mitochondrial biogenesis leading from chronic ketosis (Bough et al., 2006). Therefore, intracellular ATP is a promising mechanism for mimicking the ketogenic diet *in vitro*. From these reports, reducing and increasing extracellular glucose and intracellular ATP, respectively, might in combination reproduce ketogenic diet conditions in acute hippocampal slices (Figure 2). As mentioned in “Advantages of the Hippocampal Slice Preparation in Studying Antiepileptic Mechanisms of Ketogenic Diet” Section, it is easy to change extracellular solution with an *in vitro* brain slice (including moderately lowering glucose), but trickier to change the intracellular milieu. The whole-cell patch clamp technique is one of the methods for recording a single cell (Kawamura et al., 2004). This technique allows physical exchange between the intracellular fluid and the artificial intracellular solution in the recording pipette, enabling experimental modification of the intracellular fluid of the recorded neuron (Figure 2) including elevating intracellular ATP. We recorded from CA3 pyramidal neurons with the whole-cell patch clamp technique in acute hippocampal slices from control diet-fed SD rats or C57BL/6 mice. We found that increased intracellular ATP (comparing 0.5, 1, 2 and 5 mM ATP concentration in the intracellular solutions) and reduced extracellular glucose (from 11 mM to 3 mM) caused an outward current (hyperpolarization when recording membrane potential) in CA3 pyramidal neurons (Kawamura et al., 2010). The direction and magnitude of this current was dose-dependent for both extracellular glucose and intracellular ATP, and importantly it was found in both rats and mice. Pharmacological and genetic experiments demonstrated that when intracellular ATP was sufficient or increased, reducing extracellular glucose opened pannexin-1 channels and released intracellular ATP to the extracellular space. Released ATP was rapidly hydrolyzed to adenosine which activated adenosine A_1_ receptors (A_1_R) with subsequent opening of K_ATP_ channels. Opening of these potassium channels caused hyperpolarization and reduced excitability. These results indicate that mimicking the ketogenic diet condition with increased ATP and reduced glucose reduces excitability in hippocampal CA3 pyramidal neurons with autocrine modulation via adenosine A_1_R, and this might be a one of the key mechanisms of the anticonvulsant effects of the ketogenic diet *in vivo* (Figure 4). This approach for reproducing ketogenic diet conditions in acute hippocampal slice is useful to elucidate detailed mechanisms within single neurons. However, it mimics only two of the aspects of ketogenic diet feeding. Further examinations using behavioral tests and recordings from *in vivo* ketogenic diet-fed animals are needed to link the results from this approach to the effects of ketogenic diet feeding.

### Hippocampal Slices From Ketogenic Diet-Fed Rodents

A third approach is possibly the most direct and useful way for investigating mechanisms of ketogenic diet because it uses acute hippocampal slices from ketogenic diet-fed animals (Figure 3). In patients, ketogenic diet is applied through three meals with snacks and the typical fat: (protein+carbohydrate) ratio is 3:1 or 4:1 in children (Hartman and Vining, 2007; Zupec-Kania and Spellman, 2008) and down to 1:1 in adults. On the other hand ketogenic diet for rodents is usually applied by free access to food with more strict ketogenic ratio 4.8:1 or 6:1 (da Silveira et al., 2010). One question about this approach is whether or not the intra- and extracellular milieu produced by ketogenic diet feeding can be maintained after making and incubating brain slices. However, four reports show that it can work successfully. Stafstrom et al. (1999) reported that a ketogenic diet (4.8:1 ratio) induced antiseizure effects in acute hippocampal slices from kainic acid-treated rats. They recorded fEPSP and PS from area CA1 from SD rats fed a ketogenic diet for 6–8 weeks. Synaptic transmission was not significantly different between slices from control diet-fed and ketogenic diet-fed rats. However the frequency of kainic acid-induced spontaneous seizures was significantly lower in slices from ketogenic diet-fed rats than from control diet-fed rats. The slices were incubated by normal artificial cerebrospinal fluid not including ketone bodies. The authors concluded that the effects observed in the slices from ketogenic diet-fed rats would be independent of direct ketone body action (Stafstrom et al., 1999). Similar results were reported by Simeone et al. (2014) using extracellular multielectrode array recordings in acute hippocampal slices from ketogenic diet-fed *Knca1*-null mice. The pathologic seizure-like events generated in *Knca1*-null mice slice were diminished by ketogenic diet (6:1 ratio) treatment for 11–15 days. Mossy fiber-CA3 dendritic field potential slopes and fiber volley amplitudes of mossy fiber were not significantly different between slices from control diet-fed and ketogenic diet-fed *Knca1*-null mice, however ketogenic diet increased paired-pulse facilitation ratios and the half maximal stimulation intensities of field potential slope which are decreased by Kv1.1 knock out. They suggested that the improvement of mitochondria function by ketogenic diet might restore the hyperexcitability of CA3 neurons in *Knca1*-null mice and decrease seizure-like events (Simeone et al., 2014). Bough et al. (2006) recorded medial perforant pathway-evoked fEPSPs from the dentate molecular layer in acute hippocampal slices from SD rats fed with a ketogenic diet (6:1 ratio) for over 20 days. Reducing extracellular glucose concentration from 10 to 2 mM depressed the slope of fEPSP reversibly in slices from control diet-fed rats and this depression was inhibited in slice from ketogenic diet-fed rats. The effects were lost after slices were incubated in 10 mM glucose for over 3.5 h. This result, however, strongly suggests that intracellular metabolic changes with ketogenic diet can be maintained over 3 h after changing extracellular conditions into artificial cerebrospinal fluid. They concluded that synaptic transmissions in hippocampal slices from ketogenic diet-fed rats were more resistant to reduced glucose than slices from control diet-fed rats with facilitation of mitochondrial biogenesis (Bough et al., 2006). We also reported that ketogenic diet caused antiseizure effects in acute hippocampal slices of rats and mice (Kawamura et al., 2014). We recorded PS and GABA receptor blocker bicuculline-induced seizure-like bursting from the CA3 region in acute hippocampal slices from SD rats or C57BL/6 mice fed a ketogenic diet (6:1 ratio) for 13–18 days. Excitability and bicuculline-induced bursting were significantly inhibited by reduced extracellular glucose concentration in slices from ketogenic diet-fed rats and mice but were not changed by reduced extracellular glucose in slices from control diet-fed rodents. In this study, the effect of ketogenic diet feeding is maintained for at least 6 h after making hippocampal slices. Ketogenic diet-induced suppression of bicuculline-induced bursting was inhibited by adenosine A_1_R antagonist and did not occur in slices from adenosine A_1_R knock-out mice. Antagonism of K_ATP_ channels or pannexin-1 channels inhibited the ketogenic diet-induced suppression of bicuculline-induced bursting. These results suggest that ketogenic diet causes antiseizure effects through a pannexin-1 channel-adenosine A_1_R-K_ATP_ channel autocrine pathway (Figure 4), the same pathway revealed by the whole-cell patch clamp technique for mimicking ketogenic diet conditions as described in “Changing Intracellular ATP and Extracellular Glucose with Whole-Cell Patch Clamp to Mimic Ketogenic Diet” Section (Kawamura et al., 2010).

These studies used acute hippocampal slices from ketogenic diet-fed rodents successfully to elucidate altered neuronal activity underlying this treatment. Interestingly, reducing extracellular glucose concentration is thought be one of the most important points for reproducing the effects of ketogenic diet in this approach. Synaptic transmission in hippocampal slices from ketogenic diet-fed rodents were not different from slices from control diet-fed rodents when extracellular glucose concentration in artificial cerebrospinal fluid is standard in all three reports [however, evidence is mounting that this standard glucose concentration for acute brain slices is higher than physiological brain glucose levels (Shram et al., 1997; Lowry and Fillenz, 2001; Kealy et al., 2013)]. Reduced glucose reveals the difference between ketogenic diet- and control diet-fed animals in two of these studies (Bough et al., 2006; Kawamura et al., 2014), which parallels the finding that the anticonvulsant effect of the ketogenic diet is correlated with plasma glucose levels (Mantis et al., 2004). Therefore it would be useful to make extracellular glucose concentrations lower than standard to reproduce or maintain effects of the ketogenic diet in acute hippocampal slices.

## Conclusion

Here we describe three approaches for researching anticonvulsant mechanisms of ketogenic diets by using electrophysiological recording from hippocampal slices. The usefulness of hippocampal slices is that it is easy to elucidate the details of neuronal modulation by ketogenic diet as shown in Figure 4. All three approaches have contributed to finding detailed potential mechanisms underlying ketogenic diet effects including VGLUT modulation, K_ATP_ opening, activation of adenosine receptors, and ATP release from pannexin channels. Complementary *in vivo* work has provided additional evidence for some of these mechanisms (Masino et al., 2011; Giménez-Cassina et al., 2012). Taken together, it is clear that electrophysiological recordings from hippocampal slices is a good tool for ketogenic diet research. However, all three approaches need experimental manipulations for reproducing ketogenic diet effects *in vitro* such as ketone application, reduced extracellular glucose and increased intracellular ATP, and this requirement may explain the lack of a standardized protocol and a robust literature in this area. Finally, electrophysiology in slices is not a direct measurement of the *in vivo* anticonvulsant effects of ketogenic diet, and a combination of both *in vivo* and *in vitro* recordings is the best approach to provide further insight into the key anticonvulsant mechanisms underlying ketogenic diet and other metabolic therapies.

## Author Contributions

MK, DNR and SAM wrote the article.

## Conflict of Interest Statement

The authors declare that the research was conducted in the absence of any commercial or financial relationships that could be construed as a potential conflict of interest.
